# Does Syntactic Alignment Effectively Influence How Speakers Are Perceived by Their Conversation Partner?

**DOI:** 10.1371/journal.pone.0153521

**Published:** 2016-04-15

**Authors:** Lotte Schoot, Evelien Heyselaar, Peter Hagoort, Katrien Segaert

**Affiliations:** 1 Max Planck Institute for Psycholinguistics, Nijmegen, The Netherlands; 2 Donders Institute for Brain, Cognition and Behaviour, Nijmegen, The Netherlands; 3 School of Psychology, University of Birmingham, Birmingham, United Kingdom; Max Planck Institute for Human Cognitive and Brain Sciences, GERMANY

## Abstract

The way we talk can influence how we are perceived by others. Whereas previous studies have started to explore the influence of social goals on syntactic alignment, in the current study, we additionally investigated whether syntactic alignment effectively influences conversation partners’ perception of the speaker. To this end, we developed a novel paradigm in which we can measure the effect of social goals on the strength of syntactic alignment for one participant (primed participant), while simultaneously obtaining usable social opinions about them from their conversation partner (the evaluator). In Study 1, participants’ desire to be rated favorably by their partner was manipulated by assigning pairs to a Control (i.e., primed participants did not know they were being evaluated) or Evaluation context (i.e., primed participants knew they were being evaluated). Surprisingly, results showed no significant difference in the strength with which primed participants aligned their syntactic choices with their partners’ choices. In a follow-up study, we used a Directed Evaluation context (i.e., primed participants knew they were being evaluated and were explicitly instructed to make a positive impression). However, again, there was no evidence supporting the hypothesis that participants’ desire to impress their partner influences syntactic alignment. With respect to the influence of syntactic alignment on perceived likeability by the evaluator, a negative relationship was reported in Study 1: the more primed participants aligned their syntactic choices with their partner, the more that partner decreased their likeability rating after the experiment. However, this effect was not replicated in the Directed Evaluation context of Study 2. In other words, our results do not support the conclusion that speakers’ desire to be liked affects how much they align their syntactic choices with their partner, nor is there convincing evidence that there is a reliable relationship between syntactic alignment and perceived likeability.

## Introduction

In social interaction, humans tend to imitate their partner’s posture, gestures and mannerisms, without being aware that they do so (behavioral mimicry [1]). This kind of automatic imitation does not only occur in behavioral mannerisms, but also in verbal interaction. Speakers imitate low-level linguistic features such as accents [2], speech rate [3] and speech rhythm [4], but they also repeat their conversation partner’s lexical [5] and syntactic choices [6]. The latter is called syntactic alignment [7]. Syntactic alignment is a result of a largely automatic priming mechanism [7] and therefore often explained by mechanisms of implicit learning (e.g. [8–10]), residual activation (e.g. [11]) or a combination of both (e.g. [12]). In addition, it has been proposed that syntactic alignment can function as a tool to mediate interpersonal distance [2, 13–16]. These theories suggest that the (desired) relationship between speakers in a conversation can modulate the strength of syntactic alignment. For example, speakers would show stronger syntactic alignment effects when they interact with a partner they like or want to be associated with than when they interact with a partner they do not like or want to distance themselves from [2].

Some recent studies have provided initial evidence in line with the hypothesis that the strength of syntactic alignment can be influenced by the speakers' feelings toward their conversation partner. However, it is unclear whether this effect is positive or negative: different studies report different effects. Balcetis and Dale [14], for example, let participants perform a syntactic priming experiment with a same-sex confederate. Before the start of the actual experiment, participant and confederate each responded to a set of questions. The confederate’s answers to the questions were scripted so that for half of the participants, the confederate would come across as nice and for the other half as mean. The results of a subsequent syntactic priming experiment show that participants align their syntactic choices more when they were paired with the ‘nice’ participant than when they were paired with the ‘mean’ participant.

Contrasting results come from a study by Weatherholtz, Campbell-Kibler and Jaeger [15]. They let participants listen to one out of three different speakers, each with a different accent, talking about a political issue from a specific ideological standpoint. Results from a directly following syntactic priming experiment in which participants were primed with double object (DO) or prepositional object (PO) structure, showed that participants align *less* with PO prime sentences when they perceived themselves to be more similar to the speaker. Furthermore, participants aligned less with DO prime sentences when they perceived the speaker to be smart. Hence, in this study, there is a negative effect of personality traits of the speaker that are generally considered positive on syntactic alignment.

Although conflicting, the aforementioned studies do provide some evidence in favor of the idea that the strength of syntactic alignment can be influenced by social aspects of an interaction. One possibility why results might have been conflicting is that the focus in these studies is very unidirectional: it is only investigated whether speakers' feelings about their conversation partner influence syntactic alignment. Of course, there might be a relationship between alignment and managing interpersonal distance in the opposite direction as well [2]. It may not just be the likeability of your partner *per se*, but rather also how much you want your partner to like you, which influences syntactic alignment. Although generally these two will be highly correlated (likeability of your partner may lead to a reciprocal feeling of wanting to be liked by your partner), one could imagine situations where speakers want their partner to like them, irrespective of whether they like their partner or not. This is for example the case in a job interview. Applicants may not necessarily think highly of their potential employer’s personality, but if they really want the job, they would want the employer to evaluate them positively anyway. Since neither Balcetis and Dale nor Weatherholtz et al. have explicitly manipulated the social goals of the primed participants, this might contribute to the conflicting results they have reported: maybe there is a difference between studies in how much speakers want to be evaluated positively by their partner.

In the current study, we therefore test whether the social goal to make conversation partners evaluate them favorably automatically influences the strength of speakers’ syntactic alignment, irrespective of how they feel about their partner. To our knowledge, there has only been one previous study with a similar research question. Coyle and Kaschak [16] showed that when speakers have an (unconscious) goal to make their partner like them, they tend to align less with their partner’s syntactic choices. The experimenters let heterosexual participants perform a syntactic priming experiment with a female confederate. The male participants show weaker syntactic alignment effects in response to a female confederate with a higher level of fertility (measured by the confederate’s menstrual cycle). This difference was absent for heterosexual females talking to a female confederate. Coyle and Kaschak suggest that *not* aligning with your conversation partner's syntactic choice could be a way of displaying creative behavior (in this case, innovative rather than repetitive syntactic choices), which could be an attractive quality in potential mates [17].

The results reported by Coyle and Kaschak suggest that implicit social goals, such as speakers’ desire to make their partner like them, can indeed influence the strength of syntactic alignment. If that is true, we furthermore expect that the degree with which one conversation partner aligns with the second should influence how the second conversation partner feels about the first: it should influence the first participant’s perceived likeability. Therefore, in the current experiment, we do not only ask whether and how speakers adapt their syntactic alignment behavior to match their social goals, but also whether adaptation is effective: are participants’ evaluations of their conversation partners influenced by how much the partner aligns their syntactic choices with their own?

However, it is not straightforward to measure the degree of syntactic alignment for one participant while at the same time testing what effect this type of alignment has on their partner’s opinion of them. This is because in most studies in which syntactic alignment is measured, prime sentences are not provided by a naïve participant but by a confederate. Using a confederate provides the experimenter with the necessary experimental control: experimenters can ensure that the same number of primes in each condition is presented to all participants in the experiment (hereafter “primed participant”). However, to answer the research question presented above, we cannot include a confederate in our paradigm. A confederate would be aware of the experimental manipulation and therefore would not be able to give unbiased opinions about the primed participants. We thus need the person evaluating the primed participant to also be a naïve participant (hereafter “evaluator”). However, we still need to be able to control the behavior of this evaluator, to make sure that we present an equal number of primes in each condition to each primed participant. To combat this problem, we developed a new conversation task for two naïve participants, in which one of them (the evaluator) provides the primes for the other participant. Participants are playing a card game in which they describe photographs to each other. We solved the problem of experimental control by instructing the evaluator to read out sentences written underneath the photographs, while the other is freely describing them. This way, we can test two naïve participants and measure the degree of syntactic alignment for one while getting usable evaluations from the other, without losing experimental control.

In sum, we hypothesize that in a situation in which it is important to be evaluated positively by another person (e.g. a job interview or a first date) speakers automatically adapt how much they align with their partner's syntactic choices. Based on previous literature, however, it is unclear whether these speakers will show weaker or stronger alignment effects than speakers who feel less pressure to impress their partner. On the one hand, studies suggest a positive influence of likeability of the partner on the strength of syntactic alignment [13,14] while on the other hand, others have reported that the more speakers like or want to be liked by their partner, the *weaker* their syntactic alignment magnitude [15,16]. Crucially though, we expect that if it is the case that speakers who feel more pressure to impress their partner are more likely to align their syntactic choices with their partner's structures, their partners will also evaluate them more favorably when they show stronger priming effects, and vice versa. In other words, we test whether syntactic alignment is actually an effective way to make your conversation partner like you.

In the experiments described below, we always paired two naïve participants. One of them is assigned the role of the primed participant, the other the evaluator. The primed participant freely describes photographs with active or passive sentences, whereas the evaluator reads out sentences that are written underneath the photographs (unknown to the primed participant). For the primed participants, we expect a syntactic priming effect for passive primes: we expect that participants are more likely to produce passive descriptions when their partner has produced a passive sentence to describe the previous photograph than after baseline trial—a sentence with an intransitive verb. In line with other studies focusing on response tendencies in transitive sentences [18–21], we do not expect such an effect for active primes. More specifically, we expect a ceiling effect in the baseline frequency of producing actives in our native Dutch participant group [22], due to which a priming effect cannot be detected. We manipulated between-subjects whether the primed participant feels the need to be evaluated positively. In Study 1, half of the primed participants interact with a partner who they know is going to evaluate them later. As a control, the other half of the primed participants do not know their partner is going to evaluate them after the experiment. By comparing the two groups, we can test whether having the social goal to be evaluated positively influences how much the primed participants align their syntactic choices with their partner's prime structures. The evaluator will rate the primed participant before and after the experiment, allowing us to then use these ratings to assess whether syntactic alignment effectively influences likeability. Based on the results of this study, we conducted a second, follow-up study which will be introduced and described after presenting the methods and results of Study 1 below.

## Study 1

### Method

#### Participants

We tested 120 voluntary, naïve participants (mean age: 21.1 years, SD: 2.39, 27 males). Participants were always scheduled in pairs, so there were 60 pairs. Individuals in a pair did not know each other before the start of the experiment. Pairs were randomly assigned to one of two experimental contexts: *Control* or *Evaluation* (see below). For each pair in each context, one participant was randomly assigned the role of *evaluator*, providing the primes for the participant (see below). The other participant was the *primed participant*, for whom we measured syntactic priming magnitude. One participant pair in the Control context was excluded from the analyses because the testing conditions were not identical to all other pairs: there were three experimenters present, as opposed to only one experimenter for the rest of the pairs. We thus analyzed data for 29 pairs in the Control context and 30 pairs in the Evaluation context. In the Control context, there were two male-male pairs and 16 female-female pairs. There were also 11 mixed pairs; for seven of these pairs the female participant was assigned the role of evaluator. In the Evaluation context, there was one male-male pair and there were 18 female-female pairs. There were again 11 mixed pairs; for three of these pairs the female participant was assigned the role of evaluator. All participants were native Dutch speakers and were monetarily compensated for their participation. All participants gave written informed consent in accordance with the declaration of Helsinki. The study was approved by the local Ethics Committee of the Social Sciences faculty of the Radboud University (Ethics Approval Number ECG2013-1308-120).

#### Materials

The photographs used in this experiment have been described extensively elsewhere (e.g. [22]). All photographs depicted one or two actors performing an intransitive (e.g. running) or a transitive (e.g. kissing, strangling) action, respectively. Photographs were printed on individual cards. Participants each got one deck of cards. Each deck consisted of 240 unique cards; the pictures used in both decks are identical. There were 160 cards with transitive photographs. Agent and patient roles were depicted by either a pair of adults or a pair of children, always one male and one female actor. There were 40 transitive actions depicted, each one once with the male child actor, once with the male adult actor, once with the female child actor and once with the female adult actor as the agent. Transitive cards could be described with a sentence in the active or passive voice. Transitive cards functioned as primes (when described by the evaluator) or targets (when described by the primed participant). There were also 80 intransitive cards. Intransitive actions were depicted by the same actors depicted on the transitive cards and served as filler items and baseline primes.

#### Between-pairs manipulation: Control versus Evaluation context

To test whether the magnitude of syntactic alignment is influenced when participants feel the need to impress their conversation partner, we manipulated the social status of the evaluator. For the pairs assigned to the Evaluation context, we told *both* participants before the start of the experiment that one of them would take on the role of an evaluator, who would evaluate the other after the experiment was finished. Thus, for both participants, it was clear that they were not equals in this experiment. We did stress that the task would be the same for both participants (even though it was not: see below). For the other half of the participant pairs (i.e. participants in the Control context), we did not tell the primed participants anything about the evaluative component of this study, and the evaluators were told in secret (see below). In the Control context, the evaluator thus knew that their partner believed both participants to be equal. This is in contrast with the Evaluation context, where the evaluator knew that the primed participant knew he or she was going to be evaluated by the evaluator. Thus, there was a Context manipulation for primed participants as well as evaluators. Task and procedure were identical in Evaluation and Control context, but different for the evaluator and the primed participant.

#### Task & design

**Fig 1:**
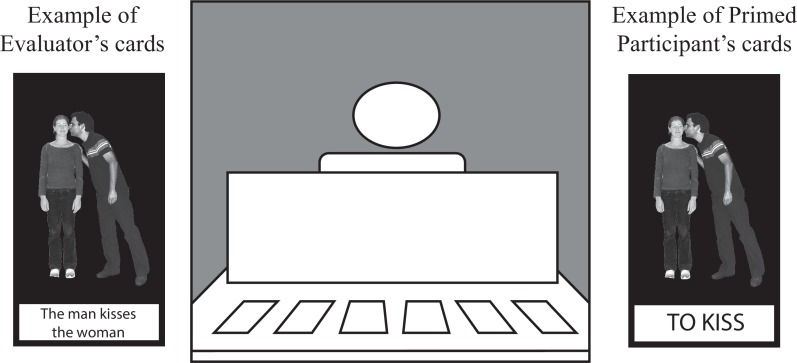
Experimental setup and materials. Middle: view for one of the participants (evaluator or primed participant). Paired participants sat across from each other at a table, with a divider between them so they could not see each other's cards. The evaluator's cards (left) showed a photograph and a description sentence. Evaluators were instructed to read out these sentences when it was their turn to describe a card. The primed participant's cards (right) had a verb written underneath the photograph. Primed participants were instructed to use this verb when they described the photograph. All materials were presented in Dutch; examples have been translated to English. Consent for publication was obtained from the actors depicted in the stimuli.

In both contexts, participants were asked to take turns describing the cards and listening to their partner's description of the cards. Each participant had their own deck of 240 cards of which any six were facing upwards at any one time. The participants’ view of their partner's set of cards was blocked by a divider (Fig 1). When it was their turn to describe a card, participants would freely pick one of the six cards in front of them to describe. This was true for both evaluator and primed participant. The partner who was listening (which switched from trial to trial) checked whether the description matched with one of their own six cards. If so, both participants removed the card and replaced it with a new one from their deck. Both participants thus always had six face-up cards in front of them. After this, the other participant would pick a card from the six face-up cards in front of them and describe it, with their partner checking whether the description matched with one of their own six cards. This turn-taking continued until all cards had been described. Since each deck consisted of 240 cards and participants took turns describing them, each partner described 120 cards. Decks were ordered identically for both partners to make sure they would not describe the same card twice.

*Primed participant*: The participants who were assigned the role of primed participant completed a free-choice syntactic priming task. When it was the primed participant's turn to describe a card, they had to describe it with a single, concise sentence, using the verb written underneath the photograph (see Fig 1: e.g. *"The man kisses the woman"* or *"The woman is kissed by the man"*). During listening trials, participants checked whether the card that was described by their partner was in the set of six face-up cards. If so, they let their partner know. Both partners then removed the card and replaced it with a new card from their deck.

*Evaluator*: The participants who were assigned the evaluator role provided the primes for the primed participant. Therefore, their cards had single, concise sentences already written out underneath the photographs (Fig 1). Evaluators were instructed to read out the sentences when it was their turn to speak. This way, we could control the number of passive and active primes that were produced by the evaluator. The cards were balanced such that 50% of the transitive sentences were in the active voice and 50% in the passive. Evaluators’ task during listening trials was the same as for the primed participant: they had to check whether the described card was in the set of six cards that were face up. Evaluators were instructed not to look at sentence structure during comprehension trials: they had to check whether one of the photographs matched the primed participant’s description, not whether the primed participant produced the exact sentence that was written underneath the photograph.

#### Questionnaires

To assess the influence of syntactic alignment on the likeability of the primed participant, we let evaluators fill in a Relationship Questionnaire. This questionnaire is based on the questionnaire used by Weatherholtz et al. [15] and consisted of 7 statements (Table 1A). Participants indicated on a 6-point Likert scale how much they agreed with the statements (1: not at all, 6: completely agree). Evaluators filled out the Relationship Questionnaire twice: once before the experiment, to measure their baseline evaluation of their partner, and once after the experiment, to see whether their partner's syntactic alignment behavior had any effect on their evaluation. Primed participants only filled out the Relationship Questionnaire once, after the experiment. It was however not the case that only the evaluator filled in a questionnaire at the start of the experiment (which would have been suspicious in the Control context). When evaluators filled in the first Relationship Questionnaire, therefore, the primed participant filled in a Conflict Questionnaire (also based on Weatherholtz et al. [15]). Again, this questionnaire consisted of 7 statements (Table 1B) and participants had to indicate on a 6-point scale how much they agreed with these statements. We aimed to use the questionnaire results obtained from the primed participants as a possible explanation for individual variation in the strength of primed participants' syntactic alignment effects (similar to [14] and [15]).

**Table 1:** Results of the Questionnaire Principal Component Analyses. (Questions presented in Dutch). Loadings greater than |0.4| are in bold as these items contribute most to the meaning of a factor. Loadings less then |0.1| are omitted for clarity.

**1A. Relationship Questionnaire**	Factor 1	Factor 2	
	Likability	Shyness	
I could be friends with my partner	**0.69**	**0.41**	
My partner is similar to me	**0.68**		
My partner appeared generous	**0.68**	0.28	
My partner intelligent	**0.68**		
My partner appeared selfish	-0.23	**-0.64**	
My partner appeared shy	-0.11	**0.83**	
My partner appeared enthusiastic	**0.72**	-0.28	
*Proportion Explained*	*0*.*63*	*0*.*37*	
**1B. Conflict Questionnaire**	Factor 3	Factor 4	Factor 5
	*Ignore*	*Dominate*	*Compromise*
I ignored the conflict and behaved as if nothing had happened	-**0.94**		
I pretended there was no conflict	**0.92**		
I tried to find a middle ground	0.14	-0.18	**0.88**
I had a discussion with the other person to try to find a middle ground	-0.28	0.22	**0.78**
I insisted that it wasn’t my fault	0.12	**0.70**	-0.16
I kept pushing until the other person saw that I was right		**0.82**	
I tried to convince the other person that my solution was the best	-0.17	**0.79**	0.16
*Proportion Explained*	*0*.*36*	*0*.*36*	*0*.*28*

#### Procedure

Both participants were picked up from the waiting room together. In the Evaluation context, participant roles were assigned randomly in the presence of the primed participant. The person sitting closest to the door would always be the evaluator. It was then explained to them that the evaluator would be evaluating the primed participant. In the Control context, role assignment information was not openly shared. The rest of the experimental procedure was identical in both conditions, but different for the evaluator and the primed participant. Both participants would first read role-specific instructions. Crucially, in both contexts, the primed participant believed they read the same instructions as their partner, which explained that they should take turns describing the photographs on the cards. However, the instructions for the evaluator explained a different task: to read out the sentences underneath the photographs. The evaluator was asked not to pose any questions about their task in the presence of the other participant. If they did have questions, they were instructed to ask the experimenter if they could go to the bathroom, which functioned as an excuse to go to the hallway with the experimenter in private. They would then get an opportunity to ask questions without the primed participant hearing them. After reading the instructions, there was a practice session (which only consisted of intransitive cards, to ensure there was no opportunity for priming), followed by both participants filling in the first questionnaire. Questionnaires were again role-specific, but the primed-participant believed them to be identical. For the evaluator, the first questionnaire was the baseline evaluation of their partner (Relationship Questionnaire 1). For the primed participant, the questionnaire consisted of statements about their conflict management strategies (Conflict Questionnaire). During the experiment, the experimenter coded both participants' utterances online for correctness (the criteria were that the agent and patient had to be named correctly and the written verb used in the sentence). Coding was later verified by another coder who was unaware of the purpose of the experiment. Only correct target responses were included in the analysis. After completing the experiment, both participants filled out the Relationship Questionnaire (second time for the evaluators). Lastly, participants were debriefed on the purpose of the experiment. None of the primed participants in the Control context were aware during the experiment that they were being evaluated. Also, none of the primed participants noticed that their partner had different cards than they did.

#### Analysis approach: Questionnaire data

Principal component analysis (PCA) was used to reduce the number of variables in the questionnaire data.

*Relationship questionnaire*: Following the advice of Reise et al. [23] on within-participant repetition of questionnaires (as was the case for the evaluators) we conducted multivariate PCA to analyze the Relationship Questionnaire. For component extraction, we combined the questionnaire responses from this experiment with data from two other syntactic priming experiments in which the exact same questionnaires were administered ([24]; Schoot et al., in prep). We then conducted multivariate PCA with orthogonal (varimax) rotation using 270 respondents (Kaiser-Meyer-Olkin Measure of Sampling Adequacy (KMO): 0.77; Bartlett’s test of sphericity: χ^2^(21): 964.64, *p* < .0001), who all filled in the questionnaire twice. We used conservative and principled criteria advocated in the statistical literature–a combination of parallel analysis and the Kaiser criterion (extract eigenvalues > 0.1)–to determine the number of factors to be extracted. These criteria indicated that a two-factor model had the greatest explanatory power for the Relationship Questionnaire data. Table 1A shows the loadings for each statement in the Relationship Questionnaire. Based on the questions with the highest loadings, we named these factors *Likeability* and *Shyness*.

*Conflict questionnaire*: For the Conflict Questionnaire we used the standard PCA with orthogonal (varimax) rotation as we did not have to account for repeated measures (KMO: 0.56; Bartlett’s test of sphericity: χ^2^(21): 489.17, *p* < .0001). Using a combination of parallel analysis and the Kaiser criterion, it was indicated that a three-factor model had the greatest explanatory power. Table 1B shows the loadings for each statement in the Conflict Questionnaire. Based on the questions with the highest loadings, we named these factors *Ignore*, *Dominate*, and *Compromise*.

#### Analysis approach: Syntactic choices primed participant

The goal of the analyses of the primed participant’s target responses was two-fold. First, it functioned as a check to see whether we could measure reliable syntactic priming effects in primed participants with our new paradigm in which we used a naïve participant instead of a confederate. Secondly, we wanted to see whether there was a difference in the degree of syntactic alignment between participants in the Control and Evaluation contexts. We analyzed the primed participant’s target responses with a generalized linear mixed effects model, using the glmer function of the lme4 package [25] in R [26]. Three conditions were included in the analysis under the factor *Prime*: baseline trials (intransitive prime followed by a transitive target), active priming (active prime followed by a transitive target), and passive priming (passive prime followed by a transitive target). Target responses were coded as 0 for actives and 1 for passives. We used a maximal random-effects structure [27]]: the repeated-measures nature of the data was modeled by including a per-participant and per-item random adjustment to the fixed intercept ("random intercept"). We began with a full model and then performed a step-wise “best-path” reduction procedure, removing interactions before main effects, to locate the simplest model that did not differ significantly from the full model in terms of variance explained. The full model included fixed effects for *Prime* and *Cumulative Passive Proportion* (see below) and two-way interactions between *Prime* and *Context*, *Cumulative Passive Proportion* and *Context*, and *Prime* and extracted factors (Relationship: *Likeability* and *Shyness*; and Conflict: *Ignore*, *Dominate* and *Compromise*). Since we had no *a priori* hypotheses about a gender effect on syntactic alignment in the current study, we did not include any main effects or interactions with this factor in our model. The factorial predictor *Prime* was dummy coded (all means compared to a reference group: intransitive baseline trials). For categorical predictors with two levels we used sum contrasts. All numeric predictors were centered.

#### Analysis approach: Ratings of evaluator

We secondly tested whether evaluators’ ratings of their partner were influenced by how strongly their partner aligned with their syntactic choices. To this end, we first calculated for each evaluator the difference in their score on each component extracted from the Relationship questionnaire (*Likeability* and *Shyness*), as measured before and after the experiment. Since we subtracted evaluators’ scores before the experiment from the same evaluators’ scores after the experiment, a positive difference score indicates that evaluators evaluated their partner as more likeable or more shy after the experiment.

We then calculated the degree of syntactic alignment for each primed participant (the proportion of passive targets following a passive prime minus the proportion of passive targets following a baseline prime). Together with the factor *Context* (Control vs Evaluation), the magnitude of the primed participant’s syntactic alignment effect was entered in linear regression models to predict the corresponding evaluator’s difference in evaluation of the primed participant. Two models were run, with the two difference scores (one for each of the components) as dependent variables.

### Results

The evaluators in the Control context produced on average 29.28 (SD: 8.04) baseline primes, 29.62 (SD: 4.55) active primes and 25.2 (SD: 4.11) passive primes. The evaluators in the Evaluation context produced on average 30.30 (SD: 8.29) baseline primes, 28.60 (SD: 3.94) active primes and 24.00 (SD: 4.24) passive primes. A repeated measures ANOVA shows no significant effect of *Context* and no significant interaction *Context* * *Prime* (both *p* > 0.5). There is a significant main effect of *Prime* (*F* (2,114) = 11.47, *p* < 0.001): evaluators were generally less likely to pick the cards with a passive description than the cards with an active or a baseline description. This reflects their natural preference for active sentence production in daily life. On average though, the evaluators still produced 24.6 passive primes: this was sufficient for our experimental manipulation.

#### Syntactic choices of primed participant

**Fig 2:**
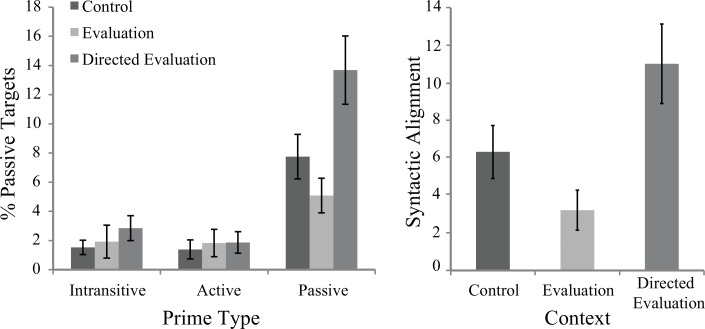
Results syntactic choices primed participants (1). Left: Average percentage of passive targets produced by the primed participants after an intransitive, active or passive prime by the evaluator, for Control, Evaluation and Directed Evaluation contexts. Right: Average degree of syntactic alignment, the percentage of passive target descriptions produced after a passive prime relative to baseline (intransitive primes), split for primed participants in the Control, Evaluation and Directed Evaluation context. All error bars represent standard errors from the mean. Note that the set-up and results of the Directed Evaluation context in Study 2 will be described and discussed in detail later on in the paper, but are depicted here for easy comparison between studies.

Our full model included the fixed factors *Context*, *Prime*, the cumulative proportion of passives produced up until that trial (*Cumulative Passive Proportion)*, and the primed participant's scores for *Likeability*, *Shyness*, *Compromise*, *Dominate* and *Ignore*. Using the step-wise ‘best path’ reduction procedure we arrived at a final model which only included fixed factors *Cumulative Passive Proportion* and *Prime*, and a random by-participants slope for *Prime*. This model was not significantly different from the full model (Full model = AIC: 1167.6 BIC: 1401.0; Best model = AIC: 1136.0 BIC: 1207.4, *p* = .8251). The results from this final mixed model are reported in Table 2. In line with previous findings in the literature, there is a significant effect of *Passive Prime* (*p* < .001): as can be seen from Fig 2 (left panel), the percentage of passive descriptions participants produced is higher for target pictures preceded by a passive prime (Control: 8.41% ± 1.61% (mean ± SE); Evaluation: 5.08% ± 1.19%) than for target pictures that were preceded by an intransitive prime (Control: 1.48% ± 0.48%; Evaluation: 1.92% ± 1.13%). Hence, there is a syntactic alignment effect for passives: participants produce more passives after passive primes relative to intransitive primes (Fig 2 (right panel): Control: 6.93% ± 1.52%; Evaluation: 3.16% ±1.05%). As expected, results show no syntactic alignment effect for actives: there were not more actives produced following active primes relative to baseline primes.

**Table 2:** Results syntactic choices primed participants in Control and Evaluation Contexts: general linear mixed effects model.

Predictor	Coefficient	*SE*	*Wald Z*	*p*
Intercept	-5.66	0.54	-10.51	< .001
Active Prime	-1.14	0.93	-1.22	0.224
Passive Prime	2.34	0.53	4.45	< .001
Cumulative Passive Proportion	1.73	1.17	1.48	.138

Note: N = 4831, log-likelihood = -557.0

**Fig 3:**
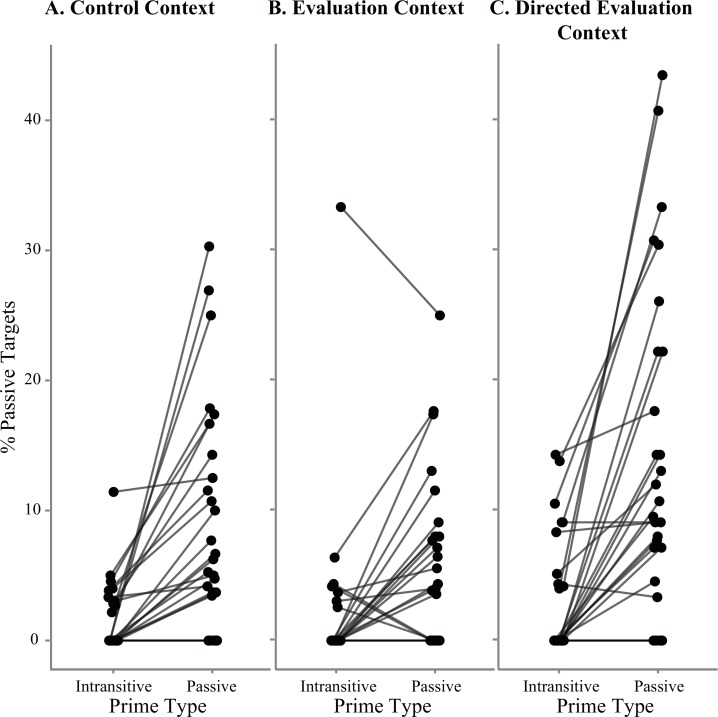
Results syntactic choices primed participants (2). Syntactic priming effect per primed participant in Control (A), Evaluation (B) and Directed Evaluation (C) contexts. For each participant, the percentage of passive targets after intransitive and passive primes is plotted. Lines connect data points from the same individual. Therefore, lines that have a positive slope indicate that participants produce more passive targets after a passive prime than after an intransitive prime: this participant shows a syntactic priming effect. Although the Directed Evaluation Context will not be discussed in Study 1, in order to allow a direct comparison between all contexts, we chose to present the data from all contexts in one figure. For more details on the Directed Evaluation context, see Study 2.

It should be noted here that the factor *Context* (Evaluation / Control) is not included in the final model. The same holds for the factors representing the primed participants' scores on the components *Likeability*, *Shyness*, *Compromise*, *Dominate* and *Ignore*. This is due to the fact that we used a step-wise "best path" model reduction procedure: these factors did not significantly improve the variance explained by the model (*p* > .05) and were therefore removed from the model. Importantly, this indicates that there was no significant effect of Context on the magnitude of syntactic alignment effects. Nevertheless, Fig 2 suggests that on average, participants in the Evaluation condition show weaker syntactic alignment than participants in the Control context. However, although standard practice, bar graphs based on averages and standard errors might not be the best way to plot group differences in this type of effects. Bar graphs obscure individual variation between participants, while linear mixed effects models do take this individual variation into account (see also [28]). Therefore, we plotted the effect of passive priming for each individual participant in each context (Fig 3, A and B). In this plot, we can clearly see that there is indeed a lot of individual variation in how susceptible participants are to syntactic priming. The difference between groups as suggested by the bar graph in Fig 2 is likely driven by just a few participants in the Control context that show a very strong syntactic alignment effect, and a few participants in the Evaluation context that show a negative effect (more passive targets after intransitive primes than after passive primes).

#### Ratings of evaluator

For each evaluator, we calculated the difference in how they evaluated their partner before and after the experiment, based on the extracted components *Likeability* and *Shyness*. Data for one evaluator in the control condition was removed because the difference score for two of the components was more than three SD above the group mean. We first ran a repeated measures ANOVA to check whether evaluators in the Control and Evaluation context differed in their initial evaluations of the primed participant. Results show that this was not the case. There is no main effect of *Context* (Control / Evaluation) and no interaction *Context* * *Component* (all *p* > .1). This means that the two groups which were sampled from the same student population were comparable before they were exposed to our manipulation.

**Fig 4:**
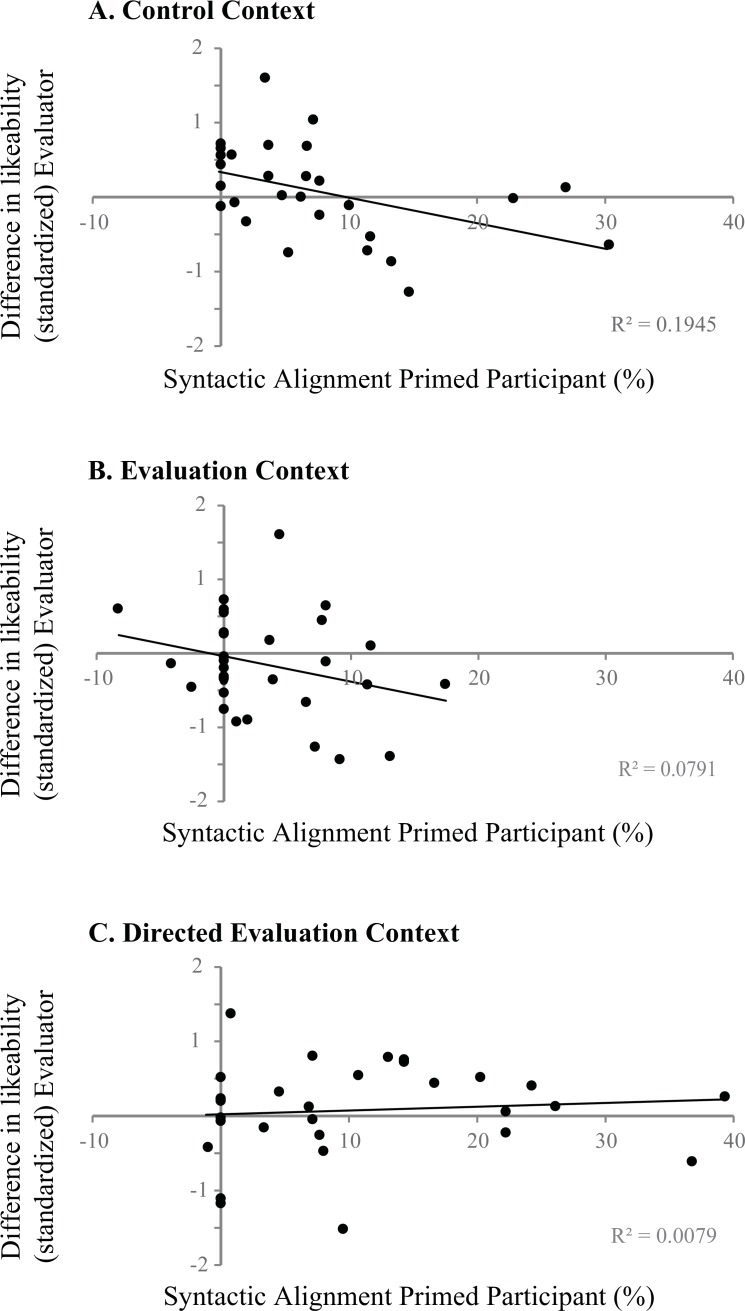
Results ratings Evaluators. The effect of the degree of syntactic alignment by the primed participant on *Likeability* as indicated by the evaluator in the Control (A), Evaluation (B) and Directed Evaluation (C) contexts. In Study 1 (Control Context (A) and Evaluation Context (B)), syntactic alignment magnitude of the primed participant has a negative effect on how the evaluator's likeability ratings of the speaker change after the experiment: evaluators decrease their rating when their partners align more with their prime structures. In study 2 (Directed Evaluation context (C)) there is no significant relationship between syntactic alignment and perceived likeability. Again, although the Directed Evaluation context is not discussed in Study 1, to allow a direct comparison between contexts, results are combined into one figure.

To test whether the difference scores (i.e. evaluation after the experiment minus evaluation before the start of the experiment) are predicted by the strength of syntactic alignment of the primed participant, we performed a linear regression analysis in R, with the evaluator’s difference score (centered) as a dependent variable. Independent variables were the *Alignment Magnitude* of the primed participant that evaluators interacted with and *Context* (Evaluation / Control). As can be seen from Table 3, for *Likeability*, we find a significant *negative* main effect of *Alignment Magnitude* (*p* < .01). As visualized in Fig 4 (A and B), the more the primed participant aligned their syntactic choices with the evaluator, the more the paired evaluator’s rating of how likeable the primed participant was decreased. In other words, syntactic alignment seems to lead to a decrease in likeability. There was no significant interaction of *Alignment Magnitude* by *Context* and there were no significant main effects or interactions for the Shyness component (all *p* > .1).

**Table 3:** Results ratings Evaluators in Control and Evaluation Contexts. Summary linear regression analysis with difference in *Likeability* (after experiment minus before experiment) ratings from the evaluator as dependent variable and *Alignment Magnitude Partner* and *Context* (Control / Evaluation) as independent variables.

	B	*SE B*	*t*	*p*
Intercept	0.15	0.11	1.43	.157
Alignment Magnitude Partner	-3.44	1.28	-2.69	.009 *
Context	0.19	0.11	1.83	.073
Context * Alignment Magnitude Partner	0.01	1.28	0.01	.996
*Adjusted R-squared*: *0*.*112*				

* Effect significant after correcting for multiple comparisons (we ran the same analyses for two outcome variables: *Likeability* and *Shyness)*

### Discussion Study 1

Study 1 was designed to investigate how participants adapt their language behavior in situations where they perceive themselves and their partner to be equals (Control context), compared to when they know they are being evaluated by their partner (Evaluation context). We assumed that in the Evaluation context, the primed participants would try to be rated favorably by an evaluator. Thus, we investigated the influence of having a social goal to make their conversation partner evaluate them positively on the degree of speakers' alignment with their conversation partner's syntactic structures. Moreover, we tested whether this is effective: does syntactic alignment actually contribute to the conversation partner's evaluation of the speaker?

To address the latter question, we let evaluators rate their partner before and after the experiment. We found that the relationship between how much primed participants aligned their syntactic choices with the evaluator's prime structures and the change in how that evaluator evaluated them on the likeability component of our questionnaire before and after the experiment was *negative*. In other words, the more the participants aligned their syntactic choices with their evaluator's prime structures, the more evaluators decreased their *Likeability* rating after the experiment (compared to before the start of the experiment). This is in line with Coyle and Kaschak's [16] suggestion that not aligning with a partner's syntactic choices may be taken as a display of creative behavior of the speaker. Although Coyle and Kaschak focus on creative behavior as an attractive quality for potential mates, based on the results of Study 1, it seems likely that displaying creative behavior (i.e. no syntactic alignment) influences the likeability of speakers in other situations as well. We argue that it is for this reason that we found a negative relationship between the primed participant's syntactic alignment magnitude and the evaluator's ratings. Indeed, the results of Study 1 alone suggest that in any context, irrespective of how explicit the evaluative component of the context is, evaluators appreciate creative language behavior more than repetitive linguistic choices.

In light of the evaluators’ results and the interpretation we have provided above, we would expect that primed participants in the Evaluation context would align less strongly with the syntactic structures of their evaluator than primed participants in the Control context. Although the bar graphs presented in Fig 2 suggest that on average, this was indeed the case, Fig 3 shows that this effect is driven by only a few participants. Indeed, we found no significant *Prime* by *Context* interaction: syntactic alignment was not stronger or weaker in the Evaluation context compared to the Control context.

However, based on these results, we cannot draw the conclusion that the desire to be evaluated positively by their conversation partner does not influence the magnitude of syntactic alignment of a speaker. Apart from the fact that any null result should always be interpreted with caution, we want to address one possible caveat of our study that might explain why we do not find any significant group level effects. We will address this issue in a follow up study described below. Of course there are other possible explanations for our null result: we will return to these in the General Discussion section of this paper.

A possible caveat of Study 1 was that although we told primed participants in the Evaluation context that they would be evaluated by their partner after the experiment ("your partner will tell us what he/she thinks about you"), we did not explicitly tell them it was important to make their partner like them. We assumed that by telling primed participants that they would be evaluated by their partner after the experiment, they would automatically and unconsciously do their best to make their partner evaluate them positively. However, there might have been individual variation between primed participants with respect to how much they valued to be evaluated positively by their partner. Perhaps group effects would have been stronger if we would have set an explicit goal for all primed participants in the Evaluation context to make their partner evaluate them *positively*. We would then expect that participants in the Evaluation context would show weaker alignment with their partner's prime structures than participants in the Control context. This would both be in line with results by Coyle and Kaschak [16] as with our finding that strong syntactic alignment has a negative effect on how likeable speakers appear to their partner. Even more, Fig 3 shows that there were a few participants in the Evaluation context that show anti-alignment (less targets described with a passive after a passive prime compared to baseline), whereas none of the participants in the Control context showed such an effect. Although purely speculative, this might mean that some participants in the Evaluation context were indeed trying harder than others to make their partner like them, but that the manipulation is not strong enough to surface at the group level.

In Study 2, we address this issue. The design of Study 2 is almost identical to the Evaluation context in Study 1, with the exception that in Study 2, we explicitly tell the primed participants to make a positive impression on their partner. Based on the results reported in Study 1, we hypothesize that if syntactic alignment is influenced by the social goals of the speaker, explicitly telling participants to make a positive impression on their partner will lead to a decrease in the magnitude of syntactic alignment, relative to the Control context reported above. By changing as little as possible to the design, Study 2 additionally allows us to test whether we can replicate the negative effect of syntactic alignment of the primed participant on the change in likeability ratings of the evaluator they are paired with.

## Study 2

### Method

#### Participants

In Study 2, we tested an additional 60 voluntary, naïve participants (mean age: 21.9 years, SD: 5.02, 12 males). All participants met the same exclusion criteria as specified in Study 1. There were 30 pairs. Individuals in a pair did not know each other before the start of the experiment. One participant pair was excluded from further analyses because the ratio of active/passive primes produced by the Evaluator was significantly different from all other pairs. We thus analyzed data for 29 pairs in Study 2. There was one male-male pair and 19 female-female pairs. There were also 9 mixed pairs; for 7 of these pairs the female participant was assigned the role of evaluator.

#### Task, design & procedure

From the perspective of the evaluators, the task, design and procedure for Study 2 were identical to the Evaluation context in Study 1. The only difference between the Evaluation context in Study 1 and Study 2 was in the instructions that were given to the primed participant. As in the Evaluation context in Study 1, both participants in the pairs in Study 2 knew that there was one evaluator who was going to evaluate the primed participant after the experiment. However, in Study 2, primed participants were presented with additional written instructions to try to make a positive impression on their partner. The evaluator was not aware of this additional task for the primed participant; therefore the instructions of the evaluator were identical to those in Study 1. We told the primed participants that the goal of the experiment was to investigate which aspects of social interaction influence how people are evaluated by their partner. Crucially, they were told that the only way to make a positive impression on their partner was in the way they described the cards—they were not allowed to engage in any type of additional verbal interaction with their partner (e.g. making jokes). From now on, we will refer to the participants who were tested in Study 2 as the participants in the ‘Directed Evaluation context’.

After completing the experiment, primed participants filled in a *post-hoc* questionnaire in which we checked whether they actually tried to make a positive impression on their partner, and if so, whether they had used a specific strategy. Crucially, all primed participants answered that they had tried to make a positive impression on their partner, but none of the participants indicated they had consciously used syntactic repetition as a strategy.

#### Analysis approach: Questionnaire data

We used the same component loadings used in Study 1 to calculate component scores for the questionnaire data of the participants in Study 2.

#### Analysis approach: Syntactic choices primed participant

For the primed participants, we compared the strength of syntactic alignment of the primed participants in the Control context, Evaluation context and the Directed Evaluation Context. (The former two were measured in Study 1 and the latter was measured in Study 2). We analyzed the primed participant’s target responses with a generalized linear mixed effects model. Model specifications remain unchanged with respect to the specifications that were reported in the Analysis Approach section for Study 1, with the exception that the predictor *Context* now has three levels. This factor was therefore dummy-coded (all means compared to a reference group: Control context). Again, we used a maximal random-effects structure and began with a full model and then performed a step-wise “best-path” reduction procedure, removing interactions before main effects, to locate the simplest model that did not differ significantly from the full model in terms of variance explained.

#### Analysis approach: Ratings of evaluator

A second goal of Study 2 was to replicate the negative effect of syntactic alignment of the primed participants on the evaluators’ rating. To this end we calculated for each evaluator the difference in their score on each component extracted from the Relationship questionnaire (*Likeability* and *Shyness*), as measured before and after the experiment and the degree of syntactic alignment for each primed participant. Together with the factor *Context* (Control / Evaluation / Directed Evaluation), the magnitude of the primed participant’s syntactic alignment effect was entered as a predictor in two linear regression models, one for each of the components as dependent variables. The factor *Context* was dummy-coded (all means compared to a reference group: Control context).

### Results

The evaluators in Study 2 produced on average 30.17 (SD: 6.82) baseline primes, 31.10 (SD: 4.90) active primes and 22.79 (SD: 4.81) passive primes. To compare these prime type ratios to the Control and Evaluation contexts, we ran a repeated measures ANOVA with between-subjects factor *Context* (3 levels) and within-subjects factor *Prime* (3 levels). Results show that there is no significant effect of *Context* and no significant interaction *Context* * *Prime* (both *p* >.5). Again, we find a significant main effect of *Prime* (*F* (2,170) = 24.87, *p* < .001): evaluators were generally less likely to pick the cards with a passive description than the cards with an active or a baseline description. This reflects their natural preference for active sentence production in daily life.

#### Syntactic choices of primed participant

Our full model included the fixed factors *Context*, *Prime*, the cumulative proportion of passives produced up until that trial (*Cumulative Passive Proportion)*, and the primed participant's scores for *Likeability*, *Shyness*, *Compromise*, *Dominate* and *Ignore*. Using the step-wise ‘best path’ reduction procedure we arrived at a final model that only included fixed factors *Cumulative Passive Proportion* and *Prime*, and a random by-participants slope for *Prime*. This model was not significantly different from the full model (Full Model = AIC: 2014.8 BIC: 2345.5; Best Model = AIC: 2023.1, BIC: 2099.1; *p* = .127). The results from this final mixed model are reported in Table 4. In line with our previous findings and other findings in the literature, there is a significant effect of *Passive Prime* (*p* < .001, Fig 2): the percentage of passive descriptions participants produced is higher for target pictures preceded by a passive prime (13.7% ± 2.29%) than for target pictures that were preceded by an intransitive prime (2.85% ± 0.83%). Hence, there is a syntactic alignment effect for passives: participants produced more passives after passive primes relative to intransitive primes. As expected, results show no syntactic alignment effect for actives: there were not more actives produced following active primes relative to baseline primes.

**Table 4:** Results syntactic choices primed participants in Control, Evaluation and Directed Evaluation contexts: general linear mixed effects model.

Predictor	Coefficient	*SE*	*Wald Z*	*p*
Intercept	-5.36	0.42	-12.63	< .001
Active Prime	-1.23	0.73	-1.69	.091
Passive Prime	2.46	0.42	5.95	< .001
Cumulative Passive Proportion	1.94	0.98	1.99	.047

Note: N = 7354, log-likelihood = -1000.6

Similar to Study 1 the factor *Context* (Directed Evaluation / Evaluation / Control) is not included in the final model. This is due to the fact that we used a step-wise "best path" model reduction procedure: the factor *Context* did not significantly improve the variance explained by the model (*p* > .05) and was therefore removed from the model. This indicates that there was no significant effect of *Context* on the magnitude of syntactic alignment effects. As can be seen from Fig 2, there does seem to be trend for more syntactic alignment in the Directed Evaluation context compared to Control and Evaluation but this is again due to only a couple of participants (Fig 3C).

#### Ratings of evaluator

Contrary to our expectations, we did not find a negative effect of *Syntactic Alignment* magnitude on the ratings of the Evaluator in the Directed Evaluation context. In the Directed Evaluation Context, the degree of syntactic alignment of the primed participants was not a significant predictor for the change in likeability rating as indicated by the evaluator they were paired with (*p* > .05). Fig 4 clearly depicts the interaction Context * Syntactic Alignment for the Likeability component (*p* = .0360, see Table 5): although there is a negative effect of syntactic alignment on perceived likeability for participants in the Control and Evaluation context, in the Directed Evaluation context this effects disappears. Syntactic alignment of the primed participant was also not a significant predictor for the difference score on the *Shyness* component of the questionnaire nor was there an effect of Context for this component (all *p* > .05).

**Table 5:** Results ratings Evaluators in Control, Evaluation and Directed Evaluation contexts. Summary linear regression analysis with difference in *Likeability* (after experiment minus before experiment) ratings from the evaluator as dependent variable and *Alignment Magnitude Partner* and *Context* (Control / Evaluation/Directed Evaluation) as independent variables.

	B	*SE B*	*t*	*p*
Intercept	0.34	0.16	2.09	.040
Alignment Magnitude Partner	-3.43	1.51	-2.28	.025
Evaluation Context	-0.38	0.21	-1.81	.075
Directed Evaluation Context	-0.32	0.23	-1.38	.171
Evaluation Context * Alignment Magnitude Partner	-0.01	2.59	-0.01	.996
Directed Evaluation Context * Alignment Magnitude Partner	3.94	1.85	2.13	.036
*Adjusted R-squared*: *0*.*064*				

### Discussion Study 2

Study 2 again showed that we can replace a scripted confederate with a naïve participant and still obtain reliable syntactic priming effects for primed participants. However, there was a crucial difference between Study 1 and 2. In Study 1, we compared the syntactic alignment magnitude of primed participants in an Evaluation context with the alignment magnitude of participants for whom the evaluator appeared to be another naïve, socially equal participant (Control context). We did not find a difference between the degree of syntactic alignment of primed participants in these two contexts. To exclude the possibility that this null result was due to individual variation in primed participants' sensitivity to the social status manipulation and their desire to be evaluated positively by their partner, in Study 2, we explicitly told the primed participants to try to make a positive impression on their partner. As in Study 1, the analysis of Study 2 did not show a significant effect of Context on the magnitude of syntactic alignment of the primed participants. However, based on a *post-hoc* questionnaire, we can be certain that all primed participants did try to make a positive impression on their partner. We can therefore exclude the possibility that the lack of a main effect of Context in Study 1 was due to the fact that there was too much individual variation between the primed participants' desires to be evaluated positively by their conversation partner. Indeed, if this were true, we should have found a significant difference between the alignment effects of primed participants in the Control context in Study 1 and the primed participants in Study 2. More specifically, based on the results of Study 1, we would have expected that participants in Study 2 would align less with their partner's syntactic choices than in the participants in the Control context. We did not find any significant results nor was there any trend in the right direction in line with the hypothesis that speakers align less with their partner in order to make a positive impression on them. Secondly, we did not replicate the negative effect of syntactic alignment on the change in ratings on the likeability component by the evaluators. There might be several explanations for this, which we will discuss in the general discussion section below.

## General Discussion

In this study, we investigated whether the degree of speakers' alignment with their conversation partner's sentence structures is influenced by having a social goal to make this conversation partner evaluate them positively. Moreover, we tested whether this is effective: does syntactic alignment actually contribute to the conversation partner's evaluation of the speaker? To be able to address both of these questions simultaneously, we developed a novel syntactic priming paradigm in which two naïve participants interacted with each other. For one of the participants, we measured syntactic alignment with different prime structures (active / passive alternation). Crucially, in our paradigm, prime sentences were not provided by a scripted confederate but by a naïve participant who read out written sentences. This way, we could achieve the experimental control that is necessary for syntactic priming paradigms but at the same time, because we let naïve participants be primers, they could also function as evaluators of the primed participants (contrary to a confederate). Below, we will first discuss the results of the primed participants, before moving on to the effect of syntactic alignment on the evaluator's rating of how likeable primed participants appeared to them.

We found reliable syntactic priming effects for primed participants in all experimental contexts. This suggests that replacing a scripted confederate with a naïve participant does not affect the basic syntactic alignment effect. Primed participants did not notice that their partner was reading out sentences instead of freely describing them, like they did themselves. As expected, we found that priming with an active transitive sentence structure does not change subsequent syntactic choices. Priming with a passive transitive sentence structure on the other hand does result in a priming effect on syntactic choices in subsequent sentences. This is consistent with the literature: priming effects for actives are found to be smaller than for passives, or absent altogether [18–21]. In fact, not only for active and passive transitives but also for many other structural alternatives, priming with the less preferred structure results in stronger syntactic priming effects (i.e. the inverse preference effect: [29–32]). Taken together, these results show that our paradigm is suited to systematically investigate the bidirectional relationship between syntactic alignment and social opinion.

In Study 1, we found no effect of context on the strength of syntactic alignment: primed participants who knew they would be evaluated by their partner (Evaluation context) did not show stronger alignment than participants who were not aware of this evaluative component (Control context). However, there was a possible caveat in the design of Study 1 that might have contributed to this null result: although we told participants in the Evaluation condition that they would be evaluated by their partner, we did not tell them it was important to be evaluated positively. Hence, we cannot be sure whether participants actually tried to make a positive impression on their partner. To exclude this possible explanation of our null-finding, we ran a follow-up study. Study 2 was identical to the Evaluation context in Study 1, with the exception that in Study 2, unknown to the evaluator, primed participants were instructed to make a positive impression on their partner. Interestingly, we again found no difference between the syntactic alignment magnitude of the participants in Study 2 and the participants in the Control context (or the Evaluation context) in Study 1. We can therefore exclude the possibility that the null finding in Study 1 was due to the fact that our context manipulation was not explicit enough.

How can we interpret the findings of the two studies together? Although null-effects should always be interpreted with caution, with 30 participant pairs tested in each group, we believe the lack of a between-context significant difference in how strongly primed participants aligned their syntactic choices with their partner's structural choices is not due to a lack of statistical power. Rather, our results seem to indicate that the degree of syntactic alignment is not automatically affected by social goals such as making your partner like you, at least not as it is manipulated in the current study. At the very least, this calls into question the robustness of the effects of social goals on syntactic alignment reported by previous studies [13–16]. Coyle and Kaschak [16], for example, found a negative effect of the speaker’s desire to impress their partner on syntactic alignment. One difference between our paradigm and the experimental design used by Coyle and Kaschak was that their manipulation of social goals was based on an intrinsic and unconscious desire of the primed participants to impress their conversation partner (i.e. mating goal), whereas our manipulation was external: we (implicitly or explicitly) tell participants to impress their partner. We cannot exclude the possibility that automatic priming effects such as syntactic alignment are only influenced when speakers are internally motivated to impress their partner: future research may investigate this issue in more detail. However, we also acknowledge the possibility that syntactic alignment might not be influenced by social goals at all. Syntactic alignment effects have been reported for participants in a non-social context, for example when primes and/or targets are presented visually in a reading paradigm (e.g. [11,33]) or when participants are producing the primes themselves [18,20]. This already indicates that syntactic alignment cannot be driven by social goals alone. Rather, there must be a more general cognitive mechanism at play, such as implicit learning (e.g. [8–10]), residual activation (e.g. [11]) or a combination of both (e.g. [12]). In this paper, we tested the hypothesis that social goals may exert a top-down influence on these automatic priming mechanisms. However, we found no evidence to support this hypothesis in the studies reported above.

The lack of a robust relationship between (desired) social relationships and syntactic alignment is also reflected in the effect of alignment on the speaker’s perceived likeability as indicated by their partner. In Study 1, we found a negative effect of syntactic alignment on how primed participants are rated by the evaluator on the likeability component of our questionnaire. This result seemed to support the hypothesis that showing creativity in linguistic choices (i.e. not aligning with a partner) is an attractive quality which leads to a positive impression of speakers [16]. However, in Study 2, we did not replicate the negative effect reported in Study 1. Considering the results of Study 1 and 2 together, then, we were thus not able to convincingly show that syntactic alignment is a reliable predictor of how likeable speakers appear to their partner. Certainly, there was no *a priori* reason to predict different results for the two studies. However, we do acknowledge that there was a trade-off between the ecological validity we achieved by including two naïve participants in the design and how much we could control their behavior. We therefore cannot exclude the possibility that primed participants in the Directed Evaluation context may have acted differently from the participants in the other two contexts, and that this may have obscured the already small effect of syntactic alignment on how likeable they were perceived by their partners. Indeed, it is likely that if there is an effect of syntactic alignment on how speakers are perceived by their partners, it will be subtle and therefore susceptible to inter-subject variation and interactions with other aspects of the conversational context.

From our post-hoc questionnaires, we have anecdotal evidence that participants in Study 2 used various strategies to make a positive impression on their partner: for example by smiling or talking with a cheerful, positive voice. It is possible that behavioral characteristics like these have interacted with the effect of syntactic alignment on perceived likeability in Study 2, leading to different results than the ones found in Study 1. However, since our study only focused on investigating the effect of syntactic alignment on perceived likeability, we can merely speculate about how between-study differences in primed participants’ behavior that are not related to syntactic choices affect how primed participants were perceived by their partner. Since these would be purely post-hoc speculations, we will not discuss them in much depth here. Instead, we would like to mention a way to address these issues in the future. By letting evaluators interact with an avatar in a virtual reality setting [34], one could control the exact behavior of the 'primed participant', varying only syntactic alignment and keeping all other behavior constant. However, although the use of avatars would allow investigators to zoom in on the effect of syntactic alignment on social opinion, making sure that any difference in social evaluation is in fact due to a difference in syntactic alignment magnitude alone, such a set-up necessarily requires experimenters to control the avatar's syntactic choices and their alignment effect. To the best of our knowledge, there have been no studies in which the alignment effect of the primed participant is manipulated. This is not surprising: it would be very hard to manipulate alignment behavior in such a way that it appears natural. More research would be necessary to decide whether and when it is natural for the avatar to align with the participant and when not. This was the main reason why in the current study, we made use of a naive participant: to achieve a naturalistic alignment pattern.

The last point we want to address here is that we did not find an effect of how the primed participant felt about the evaluator on the degree of syntactic alignment. Others have reported such an effect, although the directionality of the results has been inconsistent [14, 15]. One difference between our study and the studies in which likeability of the partner did have an effect was that in the latter studies, the likeability of the conversation partners participants interacted with was explicitly manipulated. However, again, due to the fact that in this study, we let two naïve participants interact with each other, we had no experimental control over this factor. That is, we did not explicitly manipulate the likeability of the evaluator. Therefore, individual differences between participants with respect to how they feel about their conversation partners might not have been large enough to show a significant effect on syntactic alignment.

To conclude, we have shown that our paradigm, in which prime sentences are not provided by a scripted confederate but by a naïve participant who reads out sentences, can be used to measure syntactic alignment with active/passive prime sentences for primed participants. Crucially, the participant providing prime sentences can at the same time evaluate the primed participant. This allows us to investigate whether syntactic alignment effectively influences what the evaluators think about the primed participants. We also investigated whether the degree of syntactic alignment is influenced by having an external, social goal to positively impress your partner. We undertook this research with the aim to shed new light on the relationship between social goals and syntactic alignment: whereas previous studies have only investigated the influence of social goals on syntactic alignment, we investigated whether syntactic alignment effectively influences conversation partners' perception of the speaker. However, we were not able to demonstrate an effect of social goals on syntactic alignment and our results do not provide convincing evidence that there is an effect of syntactic alignment on perceived likeability. The high ecological validity of our set-up may have contributed to the latter: we cannot exclude the possibility that there is an effect of syntactic alignment on perceived likeability, but that this effect interacts with other aspects of social behavior which we could not control for in our design. It is clear that the relationship between syntactic alignment and perceived likeability is a complex one. We here aimed to contribute to this field by developing a new paradigm and focusing on a specific and novel aspect of the research question, namely whether syntactic alignment effectively influences conversation partners' perception of the speaker. We expect that many more research observations, with large sample sizes like ours, will be needed to make a sizeable contribution to solving the complex puzzle and in the process come to a full understanding of the relationship between syntactic alignment and likeability as well as the mechanisms governing this relationship.
